# Phytochrome B Positively Regulates Red Light-Mediated ER Stress Response in *Arabidopsis*

**DOI:** 10.3389/fpls.2022.846294

**Published:** 2022-02-23

**Authors:** Gyeongik Ahn, In Jung Jung, Joon-Yung Cha, Song Yi Jeong, Gyeong-Im Shin, Myung Geun Ji, Min Gab Kim, Sang Yeol Lee, Woe-Yeon Kim

**Affiliations:** ^1^Division of Applied Life Science (BK21 Four), Institute of Agricultural and Life Science, Research Institute of Life Science, Gyeongsang National University, Jinju, South Korea; ^2^National Institute of Wildlife Disease Control and Prevention (NIWDC), Ministry of Environment, Gwangju, South Korea; ^3^College of Pharmacy and Research Institute of Pharmaceutical Science, Gyeongsang National University, Jinju, South Korea; ^4^Division of Applied Life Science (BK21 Four), Plant Molecular Biology and Biotechnology Research Center, Gyeongsang National University, Jinju, South Korea

**Keywords:** light signaling, phytochrome B, plant ER stress, red light-mediated plant growth, UPR signaling pathway

## Abstract

Light plays a crucial role in plant growth and development, and light signaling is integrated with various stress responses to adapt to different environmental changes. During this process, excessive protein synthesis overwhelms the protein-folding ability of the endoplasmic reticulum (ER), causing ER stress. Although crosstalk between light signaling and ER stress response has been reported in plants, the molecular mechanisms underlying this crosstalk are poorly understood. Here, we demonstrate that the photoreceptor phytochrome B (phyB) induces the expression of ER luminal protein chaperones as well as that of unfolded protein response (UPR) genes. The *phyB-5* mutant was less sensitive to tunicamycin (TM)-induced ER stress than were the wild-type plants, whereas *phyB*-overexpressing plants displayed a more sensitive phenotype under white light conditions. ER stress response genes (*BiP2* and *BiP3*), UPR-related bZIP transcription factors (*bZIP17*, *bZIP28*, and *bZIP60*), and programmed cell death (PCD)-associated genes (*OXI1*, *NRP1*, and *MC8*) were upregulated in *phyB*-overexpressing plants, but not in *phyB-5*, under ER stress conditions. The ER stress-sensitive phenotype of *phyB-5* under red light conditions was eliminated with a reduction in photo-equilibrium by far-red light and darkness. The N-terminal domain of phyB is essential for signal transduction of the ER stress response in the nucleus, which is similar to light signaling. Taken together, our results suggest that phyB integrates light signaling with the UPR to relieve ER stress and maintain proper plant growth.

## Introduction

Light is essential for plants as an energy source as well as a critical signal that influences plant developmental processes, including germination, de-etiolation, phototropism, vegetative growth, and reproductive development, during the entire lifecycle (Kami et al., 2010). Upon exposure to light, plants undergo photomorphogenesis, which entails cotyledon opening, leaf expansion, and shoot development (Fankhauser and Chory, 1997). The response of plants to a wide spectrum of light wavelengths (from far-red to UV-B light) allows for proper development and growth (Kendrick and Kronenberg, 1994). Plants are receptive to red/far-red light through phytochromes; blue light *via* cryptochromes, phototropins, and ZEILUPE (ZTL)/FLAVIN-BINDING KELCH REPEAT F-BOX1 (FKF1)/LOV KELCH PROTEIN2 (LKP2), and to UV-B light *via* UV-B RESISTANCE8 (UVR8; Mawphlang and Kharshiing, 2017).

Phytochromes are synthesized in the inactive Pr (red absorbing) form and are converted to the active Pfr (far-red absorbing) form upon light absorption. A high R:FR ratio increases the photo-equilibrium, that is, the ratio of active form (Pfr) to total phytochrome (Pr + Pfr), whereas the photo-equilibrium is decreased under a low R:FR ratio condition (Legris et al., 2019). Some environmental factors, such as far-red light, darkness, and high temperatures, accelerate reversion in the Pr form (Legris et al., 2016, 2019). In *Arabidopsis*, five phytochromes (phyA to phyE) have been identified and classified into two types according to the triggered responses under specific light conditions (Kircher et al., 2002; Franklin and Quail, 2010). PhyA is a type I phytochrome that is light-labile and recognizes prolonged far-red light (FR high irradiance response, FR-HIR) and very low fluence response (VLFR), as occurs in soil and deep shade. Type II is composed of phyB to phyE, which are light-stable; phyB is the most abundant than phyC-phyE (Sharrock and Clack, 2002). Type II phytochromes are activated under relatively high red/far-red ratio (FR low fluence response, LFR) light conditions and red light-dependent high irradiance responses (R-HIRs; Shinomura et al., 1996; Hirschfeld et al., 1998). Because of these differences, the action spectra are different, although the absorption spectra of types I and II are similar (Eichenberg et al., 2000; Klose et al., 2015). The far-red light-induced leaf senescence is regulated by antagonistic roles of phyA and phyB (Lim et al., 2018).

PhyB is the most abundant phytochrome in plants grown under normal light and is widespread in plant organs and tissues, acting to synchronize plant growth (Sharrock and Clack, 2002; Ha et al., 2018; Oh et al., 2018). In the root, shoot-driven light signals or stem-piped light activates phyB to trigger light responses (Lee et al., 2017). In addition, phyB acts as a thermosensor in temperature response (Jung et al., 2016). Thus, phyB combines temperature information with light signals in various morphogenic and adaptive responses, including photomorphogenesis, thermomorphogenesis, cold tolerance, drought tolerance, thermotolerance, and biotic stress tolerance, to maintain plant fitness (González et al., 2012; De Wit et al., 2013; Kim et al., 2021). PhyB has two domains, a photosensory domain (N-term) and kinase domain (C-term). The N-terminal domain of phyB is important for signal transduction, and the C-terminal domain attenuates phyB activity. Based on the truncated protein complementation experiment, it was well defined that the N-terminal domain of phyB can transduce the signal in the nucleus (Matsushita et al., 2003).

Plant growth and adaptation to various environmental changes depend primarily on the proper integration of light and stress response signaling. The abiotic and biotic stress responses are linked in the ER. Therefore, the ER is a key organelle to control multiple stress responses (Park and Park, 2019). The ER-resident luminal binding proteins (BiPs) play a role in maintaining ER homeostasis as protein-folding chaperones and UPR signaling transducers (Liu et al., 2007; Iwata et al., 2008). BiPs monitor the accumulation of misfolded proteins in response to excessive demands on the protein-folding capacity of the ER and communicate with the nucleus through two arms of the UPR signaling pathway in plants (Howell, 2013; Bao et al., 2019). One arm includes membrane-associated basic leucine zipper (bZIP) transcription factors, bZIP17 and bZIP28, which are transported to the Golgi and processed by site 1 and site 2 proteases to release their transcription factor domains toward the nucleus (Liu et al., 2007). The bZIP17 functions with bZIP28 in non-canonical UPR signaling under stress conditions (Kim et al., 2018). The other arm is the RNA splicing factor, inositol requiring enzyme1 (IRE1). Under ER stress, IRE1 is activated and participates in bZIP60 mRNA splicing to allow nuclear transport (Deng et al., 2011). In the nucleus, processed bZIP17, bZIP28, and spliced bZIP60 form a homodimer as well as a heterodimer to activate the ER stress response (Liu and Howell, 2010). The UPR signaling is associated with the plant development and stress response in the same manner (Bao et al., 2019).

Severe or prolonged ER stress can lead to PCD, called vacuolar cell death in the plant (Howell, 2013). The developmental and cell death domain (DCD)-containing asparagine-rich proteins (NRPs) function as signal transducers of PCD derived from prolonged ER stress. In Arabidopsis, NRP1 and NRP2 are involved in ER stress-induced PCD. Overexpression of BiP attenuates the ER stress-induced NRP1 and NRP2-mediated PCD (Reis et al., 2016). Metacaspases are cysteine proteases and are involved in the regulation of PCD. The nine metacaspase genes (*MC1* to *MC9*) are identified and show different expression patterns depending on the tissues and stress responses. Among them, *MC8* is strongly upregulated by oxidative stresses caused by UVC and hydrogen peroxide (H_2_O_2_; He et al., 2008). Light is an essential factor for plant growth and development, but it induces the production of reactive oxygen species (ROS), leading to PCD. High-light stress induces the expression of *OXI1* encoding a serine/threonine kinase, which accelerates the cell death response under ER stress conditions (Shumbe et al., 2016; Beaugelin et al., 2020).

A previous study defined that light enhances the ER stress sensitivity of plants, and the light signaling is coupled with ER stress response through (UPR) signaling in the nucleus. The molecular mechanism of crosstalk between light signaling and UPR is well characterized by the ELONGATED HYPOCOTYL 5 (HY5) as a negative regulator of UPR, which competes with bZIP28 to bind to the *BiP3* promoter (Nawkar et al., 2017). Although UPR activation is required to enhance protein-folding capacity to reduce light-induced ER stress, a positive regulator of UPR in light response has not yet been identified. Phytochromes may be one of the candidates for that. Especially, phyB plays an important role in abiotic and biotic challenges for plant environmental adaptation (Kim et al., 2021). Therefore, we hypothesized that phyB is involved in light-induced ER stress response.

## Materials and Methods

### Plant Material and Growth Conditions

*Arabidopsis* plants (Ler ecotype) were grown in a growth chamber (16 h light/8 h dark (16L/8D) cycles, 100 μmol photons m^−2^ s^−1^ of white cool fluorescent light) at 23°C on half-strength Murashige and Skoog (MS) medium containing 2% (w/v) sucrose and 0.7% (w/v) agar. For the phenotypic analysis of *phyB*, we used *phyB-5*, which is strong allele in *Ler* background (Reed et al., 1993) and *phyB-9*, which is the largely used for molecular genetic analyses. The complemented transgenic plants in *phyB-5* with the N- or C-terminal domain fused with GFP (NG, CG), NG fused with nuclear localization signal (NG-NLS), and PBG, which is transformed by *Agrobacterium*-mediated floral dip method with the fragments encoding the full length, N- and C-terminal domains of phyB were inserted between the 35S promoter and the Nos terminator of pPZP211/35S-NosT vector used in the previous study (Matsushita et al., 2003).

### ER Stress Response and Phenotype Analysis

Seeds were surface-sterilized with 30% bleach and incubated for 2 days at 4°C before being plated on media containing various TM concentrations under continuous white light (100 μmol photons m^−2^ s^−1^). For ER stress response analysis by qRT-PCR, 10-day-old typical grown plants were vacuum-infiltrated in the liquid media containing TM (500 ng/ml) at more than −0.1 MPa until the liquid was at a good rolling boil for 3 min and the vacuum was released gently. For ER stress phenotype analysis with light quality, 10-day-old typically grown plants were transferred to various light conditions; red (1 μmol m^−2^ s^−1^), far-red (1 μmol m^−2^ s^−1^), red: far-red (1:1, total 2 μmol m^−2^ s^−1^), dark treated with TM for 3 days.

### Quantitative RT-PCR

Total RNA was extracted from 10-day-old seedlings treated with or without TM using an RNeasy kit (Qiagen) according to the manufacturer’s instructions. First-strand complementary DNA (cDNA) was synthesized from 2 μg of total RNA using a RevertAid First Strand cDNA Synthesis Kit (Thermo Scientific) and used for qRT-PCR with a TOPreal qPCR PreMIX Kit (Enzynomics) and a CFX96 Touch Real-Time PCR Detection System (Bio-Rad). Gene expression values for each primer set were normalized relative to tubulin as an internal control. Relative gene expression levels were calculated using the comparative cycle threshold (ΔΔCt) method. All qPCR analyses were performed with three independent biological replicates. The primers used for qRT-PCR are listed in (Supplementary Table 1). Total RNA was extracted from 15 seedlings and three replicated experiments were statistically analyzed by GraphPad Prism (GraphPad Software, Inc., San Diego, CA, United States).

### Measuring Chlorophyll Contents

Chlorophyll contents were used as an indicator of leaf yellowing. Whole shoot parts were collected from 15 plants and soaked in 90% (v/v) acetone. The absorbance at 647 and 664 wavelengths was measured by spectrophotometry. The chlorophyll a and b were calculated with this formula: Chl a (μg/ml) = −1.98 * A_647_ + 11.93 * A_664_, Chl b (μg/ml) = 20.36 * A_647_ − 5.50 * A_664_ as previously described (Ritchie, 2006). Chlorophyll was extracted from 15 seedlings and three replicated experiments were statistically analyzed by GraphPad Prism (GraphPad Software, Inc., San Diego, CA, United States).

### Detection of H_2_O_2_ by Histochemical Staining

H_2_O_2_ accumulation in the plant cells was visualized using 3,3′-diaminobenzidine (DAB). Leaves of 20-day-old grown plants were detached and stained with DAB (1 mg ml^−1^, pH 3.8) for 4 h under white light (50 μmol photons m^−2^ s^−1^) condition at 23°C. The chlorophyll in leaves was removed by subsequent incubation in 80% (v/v) ethanol, and DAB staining signal is visualized as a reddish-brown color (Galvez-Valdivieso et al., 2009).

## Results

### Phytochrome B Is Required for ER Stress Response

**Figure 1:**
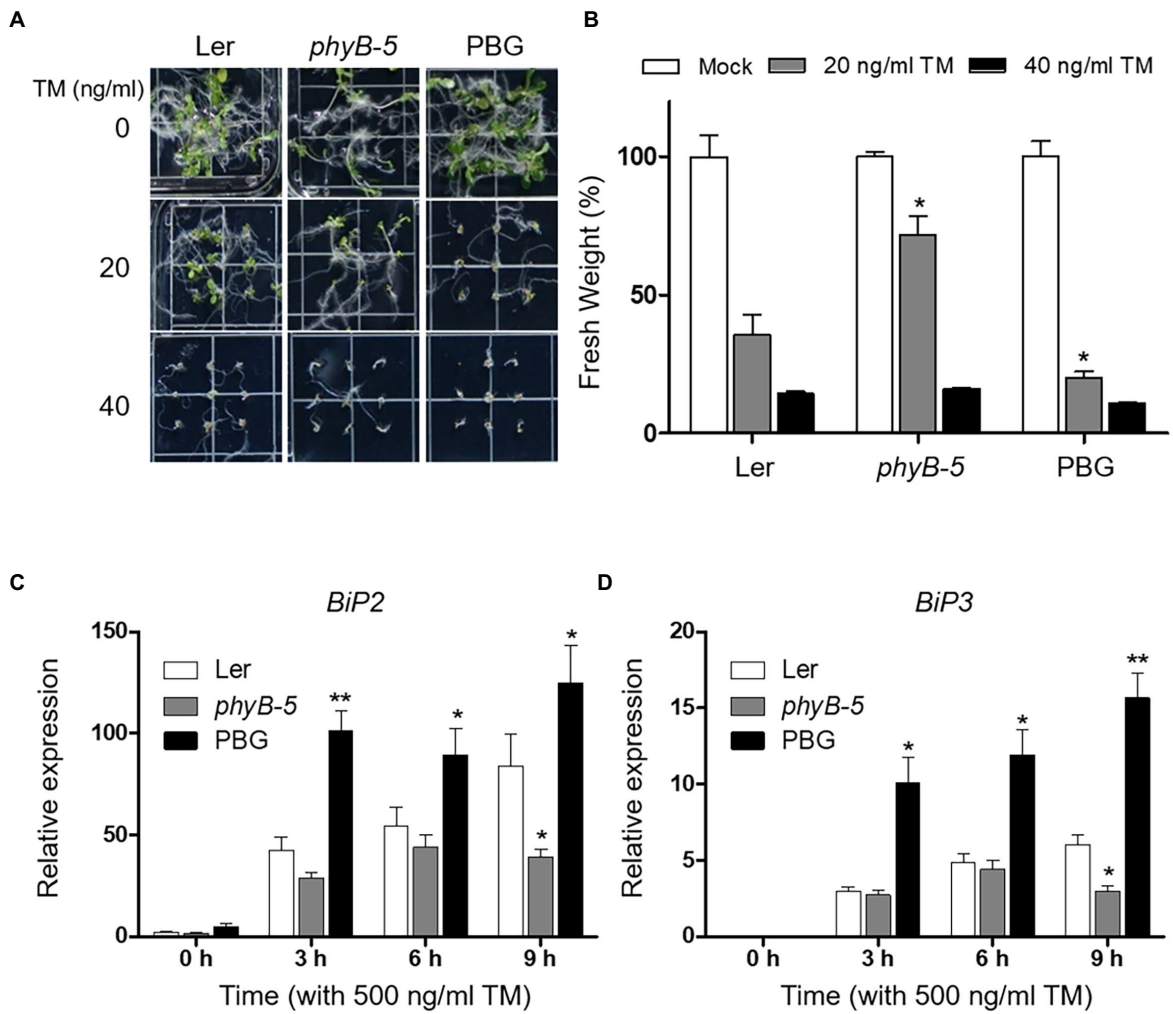
PhyB is required for endoplasmic reticulum (ER) stress rsponse. **(A)** Phenotype analysis of 10-day-old seedlings of Wild-type (Ler), *phyB-5*, and PBG (phyB-GFP overexpression) treated with tunicamycin (TM) grown under continuous white light conditions. **(B)** Relative fresh weight of shoot parts shown in **(A)**. Three biological replicates were averaged and statistically significant differences compared with WT (Ler) are indicated by asterisks (mean ± SEM; *n* = 3). **(C,D)** Transcript levels of *BiP2*, and *BiP3* were quantified using qRT-PCR. The 10-day-old seedlings of Wild-type (Ler), *phyb-5*, and PBG grown in normal media were treated with TM (500 ng/ml) by vacuum-infiltration. Three biological replicates were averaged and statistically significant differences compared with WT (Ler) are indicated by asterisks (mean ± SEM; *n* = 3). Asterisks indicate statistically significant differences (Student’s *t*-test, ^**^p < 0.01; and ^*^p < 0.05).

To test whether phyB-dependent signaling is associated with ER stress response, we observed the phenotypes of Ler, *phyB-5*, and PBG (*phyB-GFP* overexpression line) under TM-induced ER stress conditions. The *phyB-5* phenotype was insensitive to ER stress treated with 20 ng ml^−1^ TM (Figures 1A,B). Another allele, *phyB-9*, also showed the TM-insensitive phenotype (Supplementary Figure 1). In contrast, PBG showed more severe growth retardation than Ler at 20 ng ml^−1^ TM treatment condition (Figures 1A,B), indicating that phyB is involved in ER stress response in plants. Based on this phenotypic result, we can consider two possible phyB functions in ER stress response. One possibility is that PBG is sensitive to TM because PHYB acts as a negative regulator of ER stress responses. The other is that the ER stress response is highly induced in PBG because PHYB functions as an activator. Therefore, to investigate the molecular function of phyB in ER stress response, mRNA expression of UPR effector genes (*BiP2* and *BiP3*) was analyzed during TM treatment with qRT-PCR in Ler, *phyB-5*, and PBG (Figures 1C,D). Transcript levels of *BiP2* and *BiP3* were upregulated in PBG and downregulated in *phyB-5* (Figures 1C,D), indicating that phyB is a positive regulator for the ER stress response. These results suggest that phyB is essential for inducing the ER stress response.

### PhyB Contributes to the Activation of UPR Signaling

**Figure 2:**
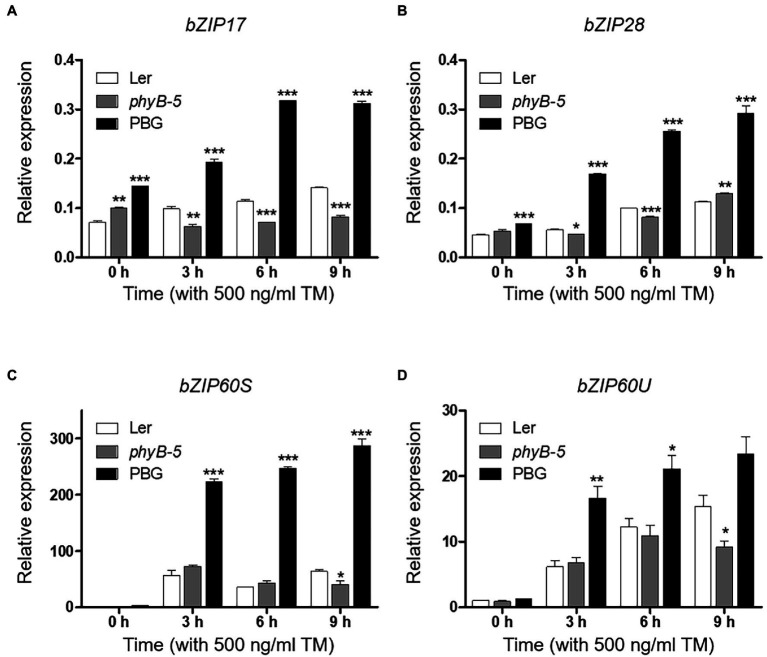
PhyB positively regulates unfolded protein response (UPR) signaling. **(A–D)** Transcript levels of UPR-related genes; *bZIP17*, *bZIP28*, *bZIP6OS*, and *bZIP6OU* were quantified using qRT-PCR. The 10-day-old seedlings of Wild-type (Ler), *phyb-5*, and PBG grown in normal media were treated with TM (500 ng/ml) by vacuum-infiltration. Three biological replicates were averaged and statistically significant differences compared with WT (Ler) are indicated by asterisks (mean ± SEM; *n* = 3). Asterisks indicate statistically significant differences (Student’s *t*-test, ^***^p < 0.001; ^**^p < 0.01; and ^*^p < 0.05).

In response to ER stress, the UPR signaling pathways are important for intracellular communication between ER and nucleus through three bZIP transcription factors (bZIP17, bZIP28, and bZIP60). To gain insight into phyB-mediated UPR signaling, mRNA levels of *bZIP17*, *bZIP28*, unspliced (*bZIP60U*), and spliced (*bZIP60S*) *bZIP60* were measured by qRT-PCR. The transcript levels of *bZIP17* and *bZIP28* were higher in PBG while lower in *phyB-5* compared to Ler (Figures 2A,B). It could be possible that phyB is associated with the induction of non-canonical UPR signaling. In addition, the major activators of canonical UPR signaling, bZIP28 and bZIP60, were highly induced in PBG and were reduced in *phyB-5* compared to Ler (Figures 2B–D), indicating that phyB-mediated signaling activates the canonical UPR signaling. Taken together, these results suggest that phyB is involved in the activation of canonical and non-canonical UPR signaling. Therefore, phyB might orchestrate the UPR pathways to maintain a balance between plant development and environmental adaptation.

### PhyB Promotes Programmed Cell Death Through Multiple Stress Responses Under ER Stress

**Figure 3:**
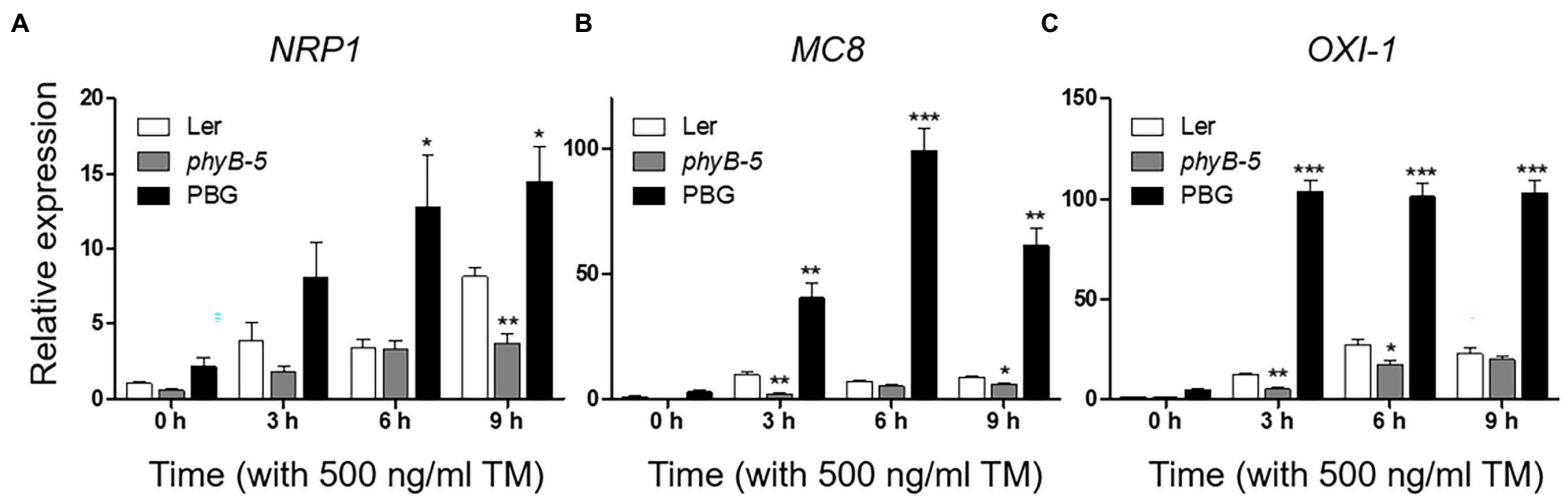
PhyB promotes programmed cell death (PCD) through ER, oxidative and light-induced stress responses under ER stress conditions. **(A–C)** Transcript levels of PCD-related genes; NRP1, MC8, and OXI-1 were quantified using qRT-PCR. The 10-day-old seedlings of Wild-type (Ler), *phyb-5*, and PBG (phyB-GFP overexpression) grown in normal media were treated with TM (500 ng/ml) by vacuum-infiltration. Three biological replicates were averaged and statistically significant differences compared with WT (Ler) are indicated by asterisks (mean ± SEM; *n* = 3). Asterisks indicate statistically significant differences (Student’s *t*-test, ^***^p < 0.001; ^**^p < 0.01; and ^*^p < 0.05).

PhyB-overexpressing plants produced a strong ER stress response and were hypersensitive to TM (Figure 1). This raised the possibility that the excessive phyB-mediated ER stress response might be correlated to PCD. To understand the effect of phyB-mediated signaling in the PCD response, we checked the expression of PCD-related genes; *NRP1*, *MC8*, and *OXI1*. The *NRP1*, which lead to PCD derived from prolonged ER stress, was highly expressed in PBG treated with TM but not in *phyB-5* plants, indicating that phyB promotes ER stress-induced PCD response (Figure 3A). The transcript levels of *MC8* were rapidly induced in PBG compared to Ler, but not in *phyB-5* plants, suggesting that phyB-mediated oxidative stress contributes to PCD enhancement (Figure 3B). *OXI1* was highly expressed in PBG compared to Ler, but not in *phyB-5* plants, suggesting that phyB-mediated light signaling participates in PCD (Figure 3C). These results indicate that phyB-mediated PCD was triggered by multiple stresses, including ER, oxidative and light-induced stress.

### PhyB Pfr Form Functions in ER Stress Relief

**Figure 4:**
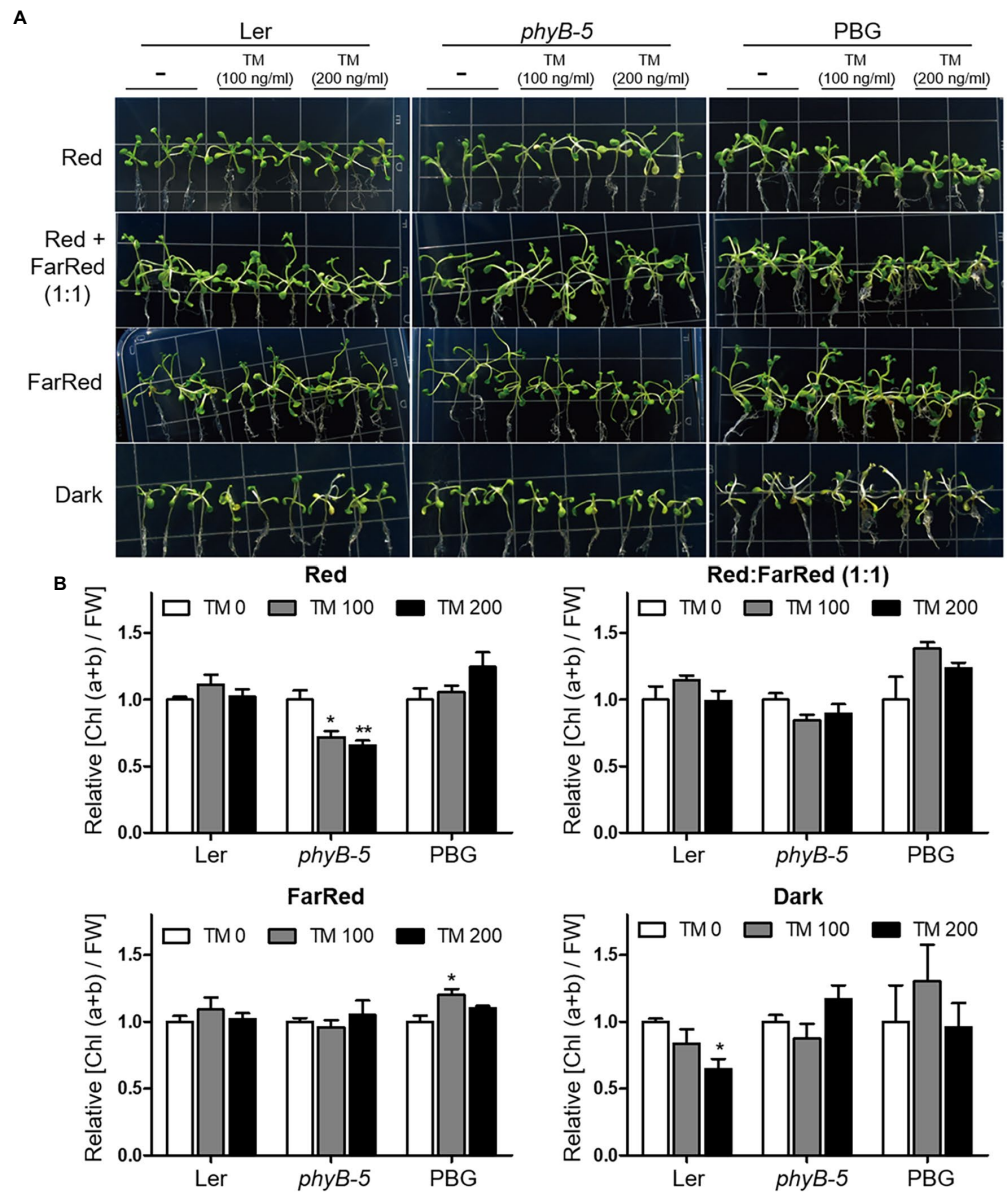
PhyB Pfr form functions in ER stress relief. **(A)** Phenotype analysis of Wild-type (Ler), *phyb-5*, and PBG (phyB-GFP overexpression) treated with tunicamycin (TM) according to light conditions. The 10-day-old seedlings of Wild-type (Ler), *phyb-5*, and PBG (phyB-GFP overexpression) grown under normal condition were transferred to TM-containing media and the Indicated light conditions; red (1 μmol m^−2^ s^−1^), far-red (1 μmol m^−2^ s^−1^), red: far-red (1:1, total 2 μmol m^−2^ s^−1^), and dark for 3 days. **(B)** Relative chlorophyll content changes shown in **(A)**. Three biological replicates were averaged and statistically significant differences compared with WT (Ler) are indicated by asterisks (mean ± SEM; *n* = 3). Asterisks indicate statistically significant differences (Student’s *t*-test, ^***^p < 0.001; ^**^p < 0.01; and ^*^p < 0.05).

The ratio of red to far-red light affects phytochrome activity. To investigate the function of phyB in the ER stress response according to photo-equilibrium, we transferred 10-day-old typical grown plants to various light conditions: red, far-red, red:far-red (1:1), and dark with TM treatment (Figure 4). Under our experimental conditions, the yellowing phenotype of leaves appeared in red light and dark conditions but not in far-red light. The observed phenotypes may be caused by ER stress related to the light response rather than leaf senescence response, because the far-red light induces a more robust leaf senescence response than darkness in Arabidopsis. Under red light conditions, *phyB-5* was sensitive to ER stress and displayed a leaf yellowing phenotype, which was not the case in the PBG (Figure 4A). Consistent with this phenotype, the chlorophyll content of *phyB-5* decreased in a TM dose-dependent manner (compared to Ler; Figure 4B). Interestingly, this phenotype was eliminated with a reduction of photo-equilibrium through far-red and dark conditions, indicating that the phyB Pfr form participates in red light-induced ER stress relief (Figures 4A,B). In contrast, *phyB-5* was resistant to ER stress under dark conditions, indicating that phyB Pr acts as an ER stress inducer. These results suggest that phyB has antagonistic roles in the ER stress response depending on photo-equilibrium.

### PhyB Transduces the ER Stress Signaling in the Nucleus Through the N-Terminal Domain

**Figure 5:**
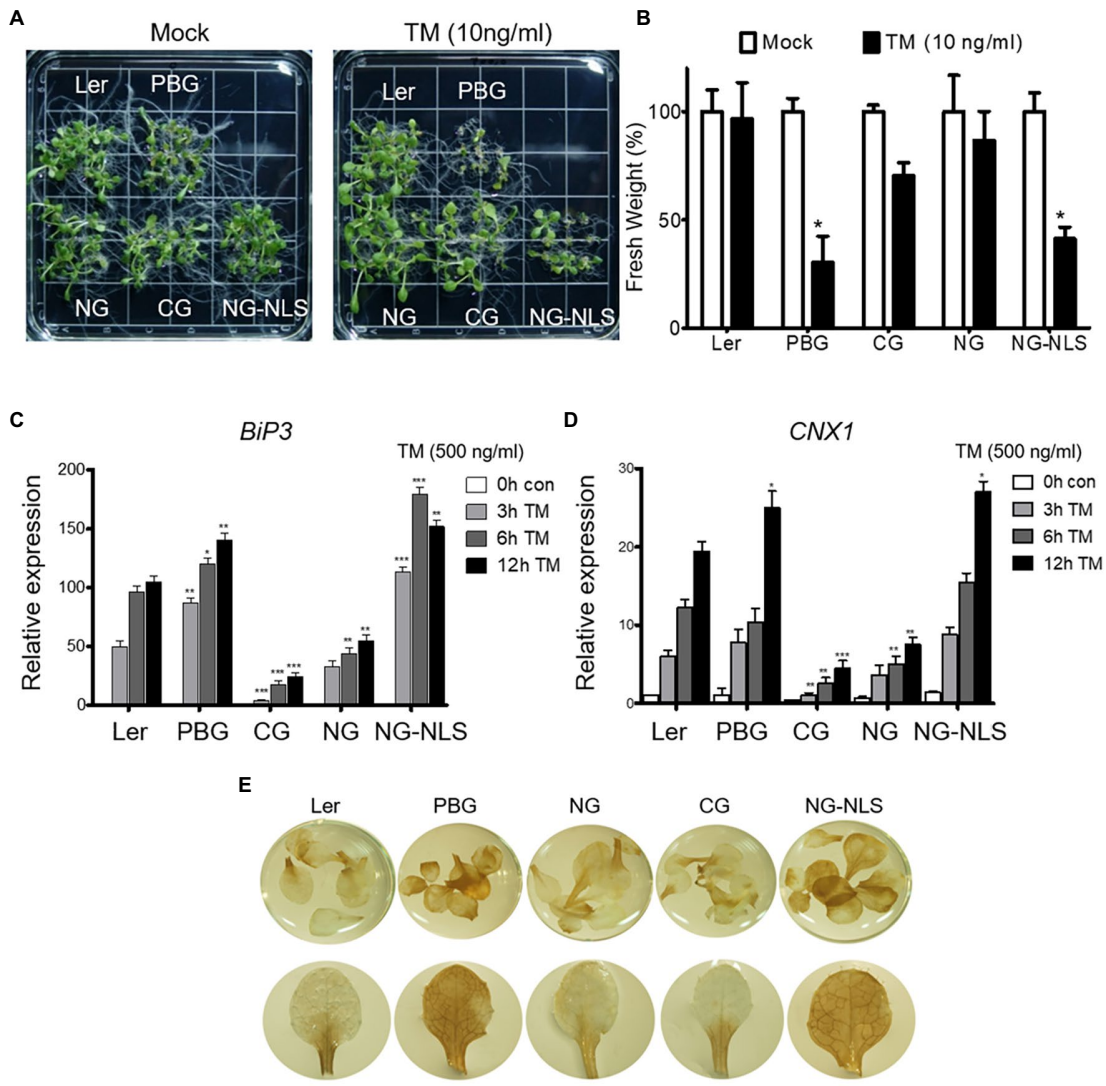
N-terminal of phyB participates in ER stress signal transduction in nucleus. **(A)** Phenotype analysis of 15-day-old plants treated with TM (10 ng/ml) grown under continuous white light conditions. **(B)** Relative fresh weight of shoot parts shown in **(A)**. Three biological replicates were averaged and statistically significant differences compared with WT (Ler) are indicated by asterisks (mean ± SEM; *n* = 3). **(C,D)** Transcript levels of BiP3, and CNX1 were quantified using qRT-PCR. The 10-day-old seedlings grown in normal media were treated with TM (500 ng/ml) by vacuum-infiltration. Three biological replicates were averaged and statistically significant differences compared with WT (Ler) are indicated by asterisks (mean ± SEM; *n* = 3). Asterisks Indicate statistically significant differences (Student’s *t*-test, ^***^p < 0.01; ^**^p < 0.01; ^*^p < 0.05). **(E)** H_2_O_2_ accumulation detected by DAB staining. Plants were grown in normal media under continuous white light for 20 days.

To understand the mechanism of phyB action in ER stress response, we examined the TM sensitivity of complemented transgenic plants expressing the N- or C-terminal domain fused with GFP (NG or CG) and NG fused with the nuclear localization signal (NG-NLS). NG-NLS plants were sensitive to TM treatment, like the PBG plants (Figures 5A,B). ER stress-inducible genes (*BIP3* and *CNX1*) were highly expressed as PBGs in NG-NLS plants (Figures 5C,D). Furthermore, ER stress causes the production of reactive oxygen species (ROS), such as free radicals and H_2_O_2_ in the cell. To further support the phenotype of NG-NLS plants, accumulation of H_2_O_2_ was visualized using histochemical DAB staining. The leaves of Ler, NG, and CG plants were found to have accumulated low amounts of H_2_O_2_, whereas abundant H_2_O_2_ accumulated in the leaves of NG-NLS and PBG (Figures 5E), indicating that the N-terminal of phyB-mediated signaling in the nucleus promotes the ROS production in the cell. PhyB was also a positive regulator to enhance the PCD-related gene expression under ER stress conditions (Figure 3). Therefore, ROS may play a key role in the regulation of phyB-mediated ER stress responses and the promotion of PCD.

## Discussion

Light is an essential factor for plant growth and development throughout life. Light signaling is closely related to ER stress response, but few studies have been conducted on it. Previous studies have shown that ELONGATED HYPOCOTYL 5 (HY5), a positive regulator of light signaling, negatively regulates UPR by competing with bZIP28 for binding to the ER stress response element (ERSE) on the promoter (Nawkar et al., 2017). Our results revealed that the red light photoreceptor phyB positively regulates the ER stress response under light conditions, unlike HY5. These results suggest that light signaling components with antagonistic functions tightly regulate the ER stress response, resulting in normal plant development.

ER is important for maintaining cellular homeostasis in cellular processes, such as plant development and environmental adaptation. Therefore, plant development and various environmental stresses affect the induction of ER stress responses. In this study, we want to understand the role of phyB with ER stress response according to photo-equilibrium. To minimize the secondary effects caused by developmental difference, 10-day-old typically grown plants were transferred to high concentration (100 and 200 ng/ml) of TM-containing media for a short time (3 days) in various light conditions: red, far-red, red:far-red (1:1), and dark (Figure 4). Under red light conditions, *phyB-5* displayed a leaf yellowing phenotype in TM dose-dependent manner, which was not the case in the PBG (Figure 4A). For quantitative phenotype analysis, we compared the change of relative chlorophyll contents per unit of fresh weight. Although chlorophyll contents were reduced in TM dose-dependent manner, their fresh weight was not significantly changed under our experiment condition (data not shown). It might indicate that plant developmental difference was minimized, and our observations were derived from the cellular process. This is consistent with the previous research showing that ER stress attributes to chlorosis without a fresh weight reduction (McCormack et al., 2015). Furthermore, leaf senescence response induces the reduction of chlorophyll content, and especially far-red light induces a more robust leaf senescence response than darkness in Arabidopsis (Lim et al., 2018). In our experimental conditions, the yellowing phenotypes of leaves were not shown in far-red light, so the leaf senescence response did not affect the phenotype. Therefore, our observations could be mainly caused by ER stress response.

The antagonistic roles of light signaling components are well defined in leaf senescence (Lim et al., 2018). Leaf senescence is accelerated by PhyB and inhibited by PhyA under far-red light conditions. Furthermore, PBG5 (*PHYB-GFP* overexpression in Col-0) showed an accelerated leaf senescence phenotype under far-red light conditions compared to Col-0 (Lim et al., 2018), indicating that phyB Pr (known as the inactive form) inhibits leaf senescence, in contrast to phyB Pfr (known as the active form). Thus, phytochromes could have two regulation modes depending on photo-equilibrium in the cellular response. The *phyB-5* plants displayed an opposite ER stress-sensitive phenotype under red light and dark conditions (Figure 4). This result supports the conception that phytochromes may also function in the Pr form, which is known as the inactive form. In *Arabidopsis*, five phytochromes (phyA to phyE) could have antagonistic functions in two forms: Pfr and Pr. Red and far-red light signaling will be fine-tuned for proper plant growth and environmental adaptation according to the photo-equilibrium of the five phytochromes.

The subcellular behavior of phyB is dynamic, that is, a translocation between the cytosol and nucleus, as well as nuclear body formation depending on environmental conditions (Legris et al., 2019). PhyB induced ER stress response genes in the nucleus *via* the N-terminal domain (Figure 5). There are two possibilities for the subcellular localization of phyB under ER stress conditions. First, the nuclear translocation of phyB may be necessary to induce the expression of ER stress response genes. Second, nuclear body formation affects the integration of light signaling and ER stress response. Thus, phyB subcellular behavior may be dynamic to integrate the light signaling and ER stress response for plant development and environmental stress adaptation.

Phytochromes regulate thermomorphogenesis as thermosensors in *Arabidopsis* (Jung et al., 2016). Recent research has revealed that a low R:FR ratio increases heat stress tolerance. PhyB inactivation, which increases the abundance of phytochrome-interacting factors (PIFs), enhances heat stress tolerance by shifting the fatty acid composition from unsaturated to saturated forms (Arico et al., 2019). In plants, high-temperature exposure leads to the accumulation of unfolded proteins in the ER, causing an ER stress response (Neill et al., 2019). Phytochromes can integrate thermosensory signaling into the ER stress response. We anticipate that understanding the integration of various phytochrome-dependent signaling pathways, including light, temperature, and different stress responses, will help overcome the challenges of growing crops in conditions introduced by global warming.

## Conclusion

**Figure 6:**
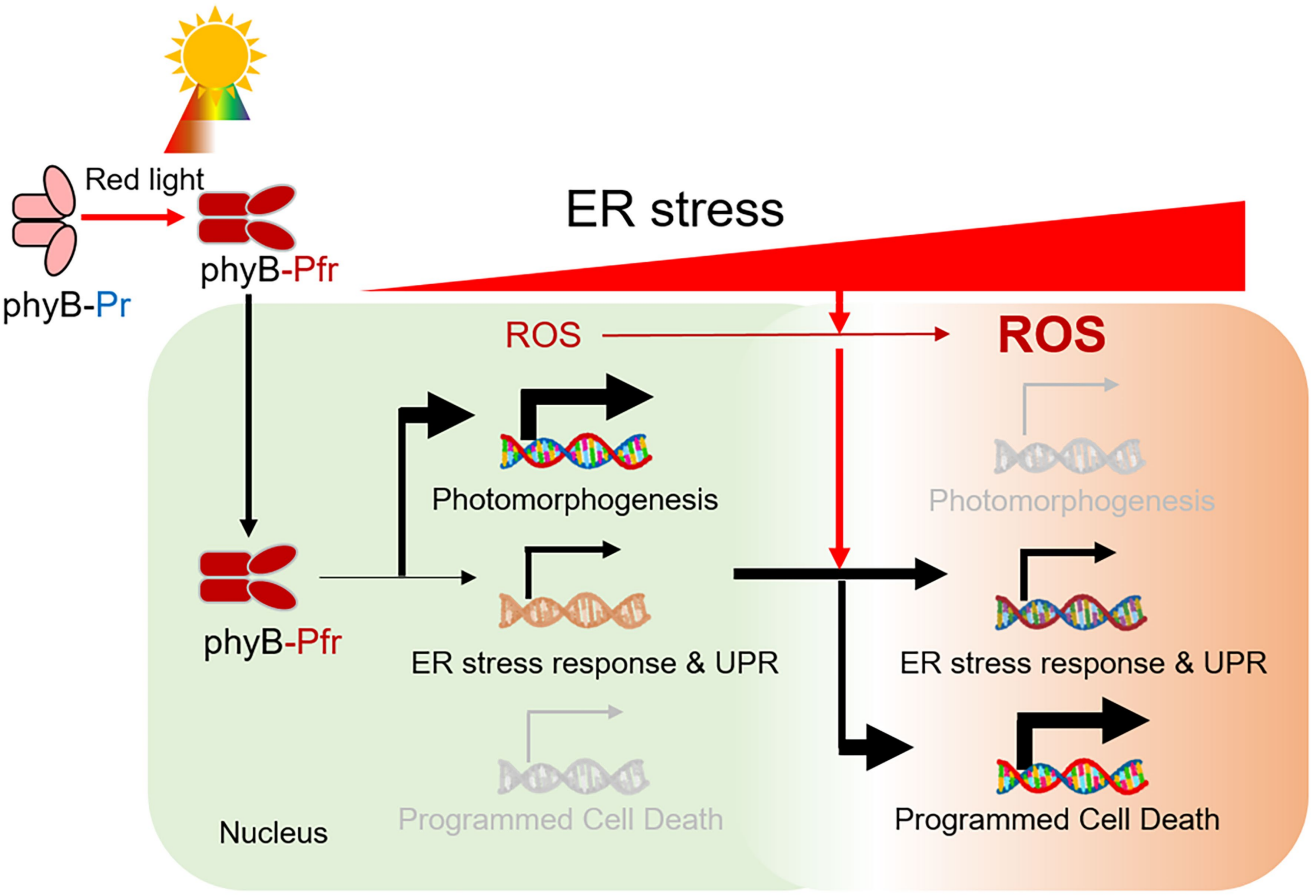
A model for the role of phyB in the red light-induced ER stress response. Red light-induced phyB Pfr form activates photomorphogenesis. During this process, phyB is required for ER stress relief by inducing an ER stress response *via* UPR signaling. In prolonged ER stress, reactive oxygen species (ROS) is highly accumulated in the cell and phyB activates ER, oxidative and light-induced stress-mediated PCD for plant survival.

In this study, we showed that phyB is required for the induction of ER stress response. The phyB-impaired mutant *phyB-5* was insensitive to TM-induced ER stress (Figure 6). On the other hand, PhyB-overexpressing plants (PBG) were sensitive to this and highly induced gene expression of the ER stress response, UPR signaling, and PCD. Interestingly, the ER stress-sensitive phenotype of *phyB-5* under red light conditions was diminished by far-red light and darkness. The N-terminus of phyB is involved in signal transduction of the ER stress response in the nucleus. Taken together, our results demonstrated that phyB integrates red light-mediated signaling with UPR in the nucleus to prevent ER stress as a positive regulator.

## Data Availability Statement

The original contributions presented in the study are included in the article/Supplementary Material, further inquiries can be directed to the corresponding author.

## Author Contributions

GA, IJ, and W-YK designed the research. GA, IJ, J-YC, SJ, G-IS, and MJ performed the experiments. GA, J-YC, MK, SL, and W-YK analyzed the data and wrote the paper. All authors contributed to the article and approved the submitted version.

## Funding

This research was supported by the National Research Foundation of Korea (NRF) grants funded by the Korean Government (MSIT-2020R1A2C3014814 to W-YK) and by Basic Science Research Program through the National Research Foundation of Korea (NRF) funded by the Ministry of Education (NRF-2021R1I1A1A01059532 to GA).

## Conflict of Interest

The authors declare that the research was conducted in the absence of any commercial or financial relationships that could be construed as a potential conflict of interest.

## Publisher’s Note

All claims expressed in this article are solely those of the authors and do not necessarily represent those of their affiliated organizations, or those of the publisher, the editors and the reviewers. Any product that may be evaluated in this article, or claim that may be made by its manufacturer, is not guaranteed or endorsed by the publisher.
