# Supplementary figures and images for: Phytochrome B Positively Regulates Red Light-Mediated ER Stress Response in Arabidopsis
Source: Front Plant Sci. 2022 Feb 23;13:846294. doi: 10.3389/fpls.2022.846294 (PMC8905361; doi:10.3389/fpls.2022.846294)

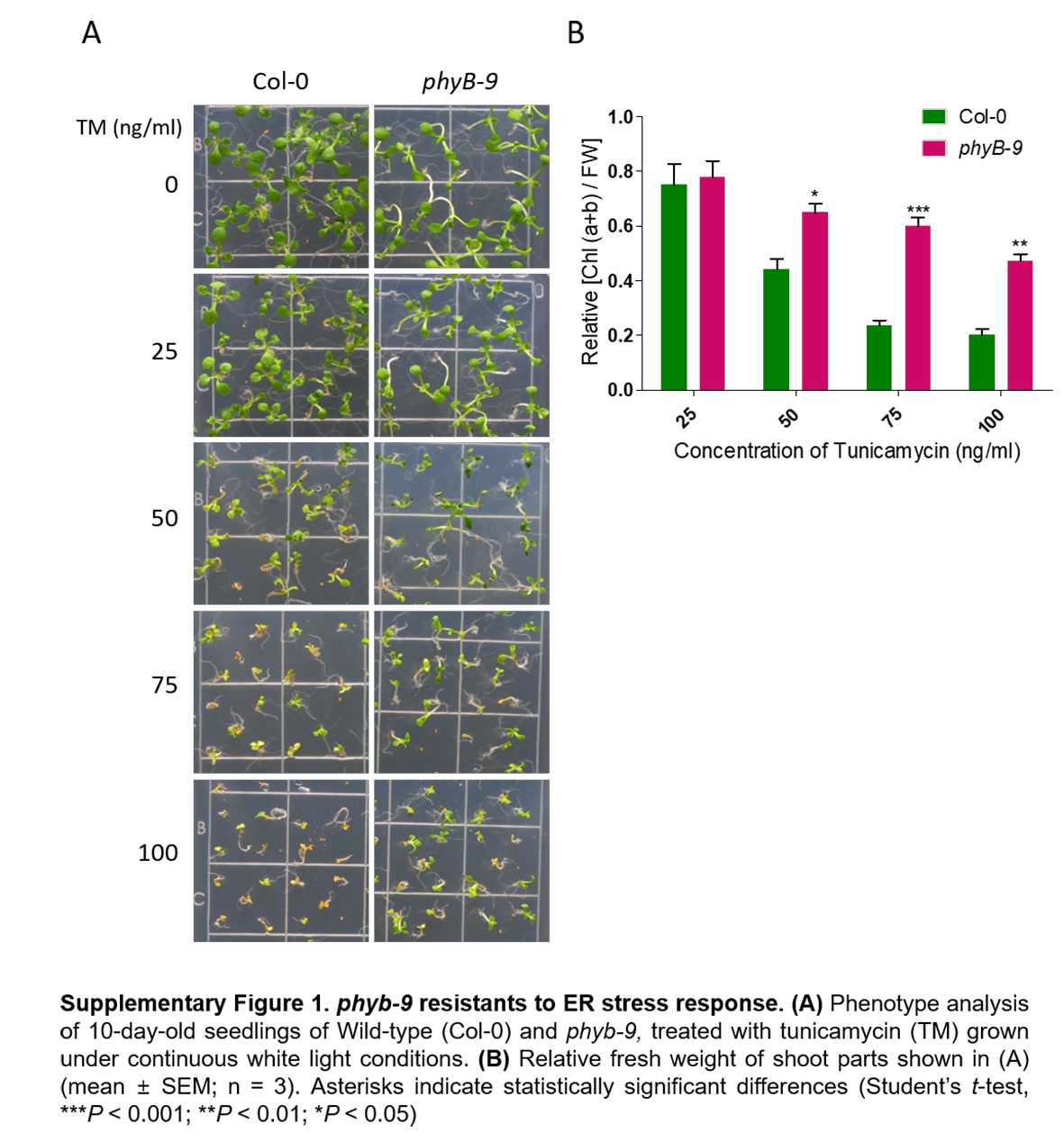

Supplement: Supplementary file 1 [file Image_1.TIF]
